# The Value of Peripheral Enhancement Pattern for Diagnosing Thyroid Cancer Using Contrast-Enhanced Ultrasound

**DOI:** 10.1155/2018/1625958

**Published:** 2018-12-02

**Authors:** Yan Zhang, Ming-bo Zhang, Yu-kun Luo, Jie Li, Zhi-li Wang, Jie Tang

**Affiliations:** ^1^Department of Ultrasound, Chinese People's Liberation Army General Hospital, China; ^2^Department of Pathology, Chinese People's Liberation Army General Hospital, China

## Abstract

**Background:**

Contrast-enhanced ultrasound (CEUS) scanning can detect differences between thyroid tumors and surrounding tissues. However, enhancement patterns within nodules are insufficient for the diagnosis of thyroid carcinomas. The peripheral enhancement patterns of nodules may provide useful diagnostic information. The objective of this study was to investigate the diagnostic accuracy of the peripheral enhancement patterns during CEUS scanning of thyroid nodules.

**Material and Methods:**

120 nodules with peripheral rings during CEUS and definite pathology confirmed by surgery were included in this study. The internal and peripheral CEUS enhancement patterns of these nodules were assessed, and the diagnostic value of CEUS was compared with the conventional ultrasound. The relationship of types of peripheral rings and sizes of nodules was analyzed, respectively.

**Results:**

There were 78 benign and 42 malignant nodules. Peripheral irregular ring performs well in detecting malignancy. It improves the diagnostic performance of CEUS by combining with internal enhancement patterns (diagnostic sensitivity of 97.6%, specificity of 98.7%, and accuracy of 98.3%) and adds value to conventional ultrasound (95.2%, 70.5%, and 79.2%). The sizes of the nodules with regular high-enhanced rings (2.34 ± 1.33 cm) were larger than the other three types of peripheral rings (*P* < 0.05).

**Conclusions:**

Features of peripheral ring on CEUS are important for the diagnosis of thyroid cancer; they can further contribute to the accuracy combining with the internal enhancement pattern, which could avoid the unnecessary biopsy.

## 1. Introduction

Since mid-1990s, the incidence of detected thyroid cancer has been increased worldwide, and it appears to be the fastest-growing cancer nowadays [1, 2]. The reason behind this phenomenon is probably the dissemination of thyroid screening. Generally, differentiated thyroid cancer is considered to be lower risk compared with other cancer. However, the clinical behaviors of this cancer are complex and varied. Some aggressive thyroid cancer has neck lymph nodal metastases, extrathyroidal invasion, and distant metastasis [3, 4]. Accurate and timely diagnosis is essential to optimize management. Although ultrasound (US) is the first-choice imaging modality for the diagnosis of thyroid cancer [5], the overlapping and similarity of some ultrasound appearances make it difficult to distinguish benign and malignant thyroid nodules. Therefore, biopsy was carried out to get the final diagnosis. Comparing with core needle biopsy [5, 6], FNA biopsy is currently the best triage test for the preoperative evaluation of the thyroid nodules. However, most (60%-80%) results of FNA biopsy undertaken were benign, and 10-15% are nondiagnostic [7], which usually result from (1) cystic or sclerotic lesions, (2) nodules with a thick or calcified capsule, (3) hypervascular or necrotic lesions, and (4) sampling errors or faulty biopsy techniques. Thus, how to improve the accuracy of preoperative diagnosis and reduce the number of unnecessary biopsies are an urgent issue that requires further research.

Angiogenesis plays an important role in tumor growth and proliferation. Unlike normal blood vessels, tumor vessels are structurally and functionally abnormal. Contrast-enhanced ultrasound (CEUS), with more sensitive imaging of tissue microvessel perfusion, has been reported to improve the identification of thyroid cancer in several studies [8–10]. Previous studies used mainly enhancement patterns and intensity for the differential diagnosis of thyroid nodules. In some studies, the enhancement patterns of solid nodules were classified as low enhancement, iso-enhancement, and high enhancement, with low enhancement considered as a malignant feature [11, 12]. In other studies, the enhancement patterns of nodules were classified into homogeneous, heterogeneous, ring enhancing, and no enhancement [13]. The results suggested that ring enhancement was a predictor of benign lesions, whereas heterogeneous enhancement was a useful indicator of malignant lesions. Although ring enhancement has been studied, the peripheral ring patterns have not been investigated in detail. Therefore, this study aimed at investigating the diagnostic performance of peripheral ring pattern in detecting thyroid malignancy.

## 2. Material and Methods

### 2.1. Subjects

This study was approved by the local ethics committee, and the participants gave written informed consent. From October 2012 to August 2015, a total of 240 patients with 253 thyroid nodules were subjected to conventional US and CEUS examination preoperatively in our hospital. Exclusion criteria were (1) patients allergic to sulfur hexafluoride microbubbles (SonoVue) or had a coagulation disorder and (2) patients underwent thyroid nodule minimally invasive ablation.

A total of 120 nodules with peripheral rings on CEUS and definite pathology confirmed by surgery were included in the final data analysis. The patients' age ranged from 14 to 69 years (43.18 ± 12.28 years). The maximum diameters of the 120 nodules ranged from 0.40 to 5.70 cm (1.90 ± 1.22 cm).

Sixty-three of the 120 nodules were surgically resected because of suspected thyroid cancer, 49 were resected because of patient requirements (palpable thyroid lump in the neck or difficulty of breathing or swallowing caused by the lump.), and eight benign nodules were resected together with other suspicious nodules.

### 2.2. Conventional Ultrasound and CEUS Examinations

An L12-5 probe of the Philips iU22 US system (Philips, Netherlands) was used to perform the US examination, with a frequency of 5–12 MHz. The L9-4 probe of the Philips iU22 US system (Philips) was used to perform the CEUS examination, with a frequency of 7 MHz and a mechanical index (MI) of 0.08.

The contrast agent used in this study was 59 mg dry powder SonoVue (Bracco S.p.A Inc., Milan, Italy) made up of 5 ml of normal saline, which was administered intravenously into an elbow vein at a dose of 2.0 ml.

Patients were scanned in the supine position with their neck extended. The size, location, boundaries, internal structures, and blood flow [14] of the lesions were assessed. The ultrasound machine was then switched to CEUS mode, and the contrast imaging was displayed side-by-side with the conventional gray-scale imaging. The real-time microbubble perfusion within the lesions and surrounding tissues was observed for a minimum of two minutes and recorded on the system's built-in hard drive.

### 2.3. Image Interpretation and Analysis

The patients were suspected of thyroid cancer by conventional US because of nodules with at least one of the following findings: hypoechogenicity, microcalcification, irregular margin, intranodular vascularity, and taller than wide [15, 16].

The recorded CEUS images were reviewed, and the enhanced intensities of thyroid nodules were classified as no enhancement (no contrast agent was found in a lesion), low enhancement (the enhanced intensity in a lesion was lower than the surrounding thyroid tissue), iso-enhancement (the enhanced intensity in a lesion was similar to the surrounding thyroid tissue), and hyperenhancement (the enhanced intensity in a lesion was higher than the surrounding thyroid tissue). When enhancement was present, it was further assessed for the degree of homogeneity, producing the following lesion enhancement categories: (1) homogeneous low enhancement, (2) heterogeneous low enhancement, (3) homogeneous iso-enhancement, (4) heterogeneous iso-enhancement, (5) homogeneous hyperenhancement, (6) heterogeneous hyperenhancement, and (7) predominantly no enhancement or the whole lesion showed no enhancement.

The peripheral rings of the nodules were divided into (1) regular high-enhancement ring, (2) irregular high-enhancement ring, (3) regular no-enhancement ring, and (4) irregular no-enhancement ring. Regular rings were round or oval, and their thickness was uniform. Conversely, irregular rings were misshapen, and their thickness was nonuniform.

Two reviewers were involved in this study, none of them did perform any examinations in this study and were hence blinded to the patients' information. They viewed the conventional US and CEUS images independently. Both reviewers were specialized in the diagnosis of thyroid disease and had performed CEUS routinely in clinical practice for more than 3 years. Discrepancies were resolved by consensus or by the judgment of a third reviewer who also specialized in CEUS.

### 2.4. Statistical Analysis

All statistical analyses were performed using SPSS software package, Version 13 for Windows (SPSS Inc., Chicago, IL, USA). A chi-squared test (*χ*^2^) was used to analyze the categorical variables. When the total observed frequency was less than 40 or the theoretical frequency was less than 1, Fisher's exact probability test was used instead of a *χ*^2^ test. Kruskal-Wallis test was used to analyze the significant difference among the means of three or more independent groups when the data did not obey the normally distributed variables and homogeneity of variance. A kappa test was used to measure the agreement between two reviewers' diagnoses. Kappa < 0.4 was considered as a poor agreement, 0.4 < kappa < 0.75 was a moderate agreement, and kappa > 0.75 was a good agreement. *P* values less than 0.05 were considered statistically significant.

## 3. Results

### 3.1. Histopathology

Seventy-eight nodules were benign, including 42 nodular goiters, 25 adenomas, 10 Hashimoto thyroiditis, and one granulomatous thyroiditis. Forty-two nodules were malignant, including 40 papillary thyroid carcinomas and two medullary carcinomas.

### 3.2. Conventional US and CEUS Findings of Thyroid Nodules

105 of the 120 nodules were classified as solid nodules and 15 as mix cystic-solid nodules on conventional US. Among the 63 nodules diagnosed as malignant by conventional US, 40 (63.5%) were malignant and 23 (36.5%) were benign, while in the 57 nodules diagnosed as benign by conventional US, 2 (3.5%) were malignant and 55 (96.5%) were benign. The diagnostic sensitivity, specificity, accuracy, positive predictive value (PPV), and negative predictive value (NPV) for thyroid cancer were 95.2%, 70.5%, 79.2%, 63.5%, and 96.5%, respectively, by conventional US.

The agreement between the two reviewers in CEUS diagnosis was high, with a Kappa index of 0.84. The internal enhancement patterns are shown in Table 1. A significant difference was found among the seven internal enhancement patterns between benign and malignant thyroid nodules (*χ*^2^ = 40.61, *P* ≤ 0.001). The diagnostic sensitivity, specificity, accuracy, PPV, and NPV for thyroid cancer were 40.5%, 92.3%, 74.2%, 73.9%, and 74.2%, respectively, according to the internal enhancement pattern of the nodules when low-enhancement pattern was considered as malignant.

**Figure 1:**
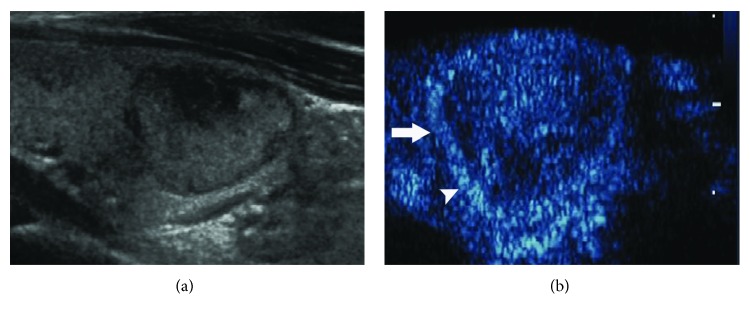
A peripheral regular high-enhancement ring of a thyroid nodule on CEUS. (a) Gray-scale US showed a heterogeneous nodule with a defined margin and a regular shape in the right lobe of the thyroid. (b) During CEUS, a heterogeneous high-enhancement pattern was found in the nodule (arrow); meanwhile, a regular high-enhancement ring (arrowhead) was displayed around the nodule. The pathology from surgery was thyroid adenoma.

**Figure 2:**
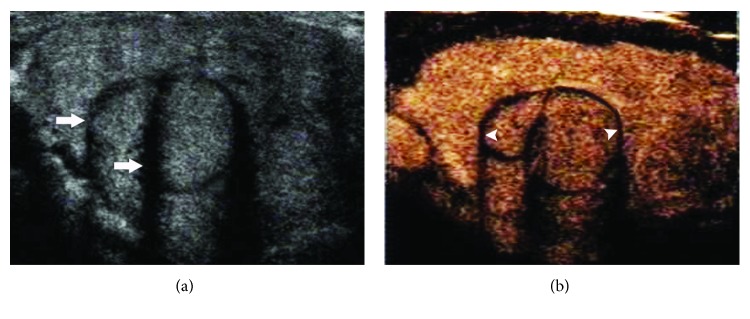
Peripheral regular no-enhancement ring of thyroid nodule on CEUS. (a) Gray-scale US showed two adjacent iso-echoic nodules with defined hypoechoic haloes and regular shapes in the left lower lobe of the thyroid (arrows). (b) During CEUS, an iso-enhancement pattern was found in both two nodules; meanwhile, regular no-enhancement rings (arrowhead) were displayed around the nodules. Both of the nodules were thyroid adenoma confirmed by surgical pathology results.

**Figure 3:**
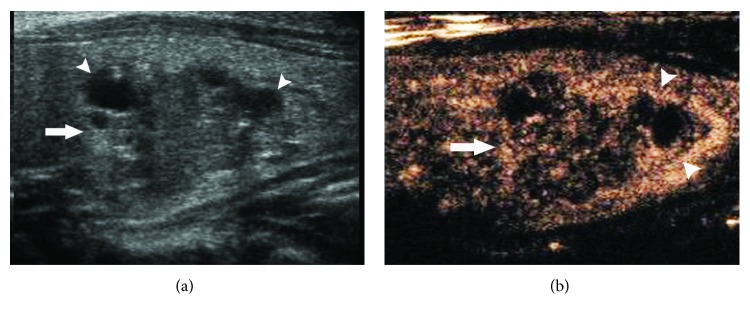
A peripheral irregular high-enhancement ring of a thyroid nodule on CEUS. (a) Gray-scale US showed a heterogeneous nodule (arrow) with an ill-defined margin, an irregular shape, and some cystic regions (arrowhead) in the left lobe of thyroid (arrow). (b) CEUS suggested a heterogeneous low-enhancement pattern (arrow) with small no-enhancing regions in the nodule and an irregular incomplete high-enhancement ring (arrowhead) around the nodule. The pathology was papillary thyroid carcinoma.

**Figure 4:**
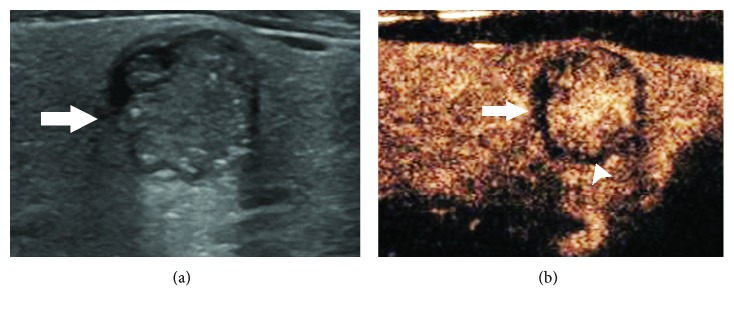
A peripheral irregular no-enhancement ring of thyroid nodule on CEUS. (a) Gray-scale US showed a hypoechoic nodule (arrow) with an ill-defined margin and an irregular shape in the left lower lobe of thyroid. (b) During CEUS, heterogeneous high enhancement was found in the nodule (arrow); meanwhile, an irregular no-enhancement ring with nonuniform thickness (arrowhead) appeared around the nodule. The pathology was papillary thyroid carcinoma.

The peripheral rings and thyroid pathological types are shown in Table 2. Regular high-enhancement rings (Figure 1) were found in 70 nodules, among which only one was medullary thyroid carcinoma, and the others were benign. Regular no-enhancement rings (Figure 2) were found in 12 nodules, including nine benign and three papillary thyroid carcinomas. There was no significant difference in regular no-enhancement rings between benign and malignant nodules (*χ*^2^ = 0.085, *P* = 0.771). Irregular high-enhancement rings (Figure 3) were found in 13 nodules, and all of them were papillary thyroid carcinoma. Irregular no-enhancement rings (Figure 4) were found in 25 nodules, and all of them were malignant, including 24 papillary thyroid carcinomas and one medullary carcinoma. Malignant criterion: (1) when a nodule had peripheral irregular ring, it was considered as malignant; (2) when a nodule had a regular no-enhancement ring, the internal low-enhancement pattern was considered as malignant. In that way, the diagnostic sensitivity, specificity, accuracy, PPV, and NPV for thyroid cancer were 97.6%, 98.7%, 98.3%, 97.6%, and 98.7%, respectively, for CEUS scanning combining internal and peripheral enhancement patterns.

The diagnostic results of conventional US and CEUS and their final pathology are shown in Table 3. Of the 25 nodules in which conventional US and CEUS have different diagnostic results, 23 nodules (92%) that were misdiagnosed as malignant by conventional US were diagnosed correctly by CEUS (20 of 23 nodules had peripheral regular high-enhancement rings).

### 3.3. Peripheral Ring Types on CEUS and Nodule Size

The sizes of the nodules were significantly different among four types of peripheral rings (Kruskal-Wallis test: *Hc* = 21.65, *P* < 0.001). The sizes of nodules with regular high-enhanced rings (2.34 ± 1.33 cm) were larger than those with irregular high-enhancement rings (1.59 ± 0.74 cm), regular no-enhancement rings (1.23 ± 0.47 cm), and irregular no-enhancement rings (1.16 ± 0.67 cm) (*P* < 0.05). No differences were found among the latter three types of peripheral rings (*P* > 0.05).

## 4. Discussion

CEUS enhancement patterns were found to be different in benign and malignant nodules, but its value in the diagnosis of thyroid cancer is controversial [11, 17–20]. Therefore, more information is needed to improve the diagnosis of thyroid cancer with CEUS. In this study, four types of rings around thyroid nodules were found, so we specially analyzed the nodules with peripheral ring signs on CEUS, and the results showed that peripheral rings contributed greatly to the diagnosis of thyroid cancer.

Our study shows that peripheral irregular rings perform well in detecting malignancy. Regular high-enhancement rings were found mainly in benign nodules, except for one case with medullary carcinoma. This medullary carcinoma was a large heterogeneous mass (5.0 × 3.9 × 4.5 cm) which showed regular shape, defined margin, and rich blood flow signal on conventional US, and internal heterogeneous iso-enhancement pattern and peripheral regular high-enhancement ring were displayed on CEUS. Those benign appearances might have led to the missed diagnosis, which suggested that the appearance of the regular high-enhancement ring is difficult to interpret for some medullary carcinomas, although this situation is rare. With the help of the peripheral enhancement patterns, the diagnostic confidence for thyroid cancer improved significantly. Compared with the criteria using only internal enhancement pattern, diagnosing thyroid cancer with combined peripheral and internal enhancement characteristics had higher sensitivity (97.6% vs. 40.5%), specificity (98.7% vs. 92.3%), and accuracy (98.3% vs. 74.2%). CEUS also performed better than conventional US, especially for the diagnostic specificity (98.7% vs. 70.5%) and accuracy (98.3% vs. 79.2%), thus could help to reduce the requirement for unnecessary biopsies. Some peripheral regular no-enhancement rings corresponded to halo signs on the gray-scale image. The presence of a halo is generally considered to be a benign characteristic [21]. However, some thin halos displayed on the gray-scale images might be unclear and hard to detect, whereas peripheral rings could be observed clearly on CEUS. This indicated that more peripheral halos could be observed by CEUS compared with conventional US. However, it should be noted that regular no-enhancement on CEUS and halos on gray-scale imaging could also be found in some malignant nodules, for example, three of the 12 nodules with regular no-enhancement rings in this study were malignant, and their internal low enhancement appearances corrected the final diagnosis. Therefore, the combination of peripheral and internal enhancement patterns is useful, especially for those nodules with regular no-enhancement rings.

The peripheral ring type might be related to the nodule size. The maximum diameter of nodules with regular high-enhancement rings was obviously larger than that of nodules with other three types of rings (*P* < 0.05). As a kind of benign tumor, solid adenomas often demonstrated expansive growth with strong tension. Thus, the adjacent thyroid tissue could be compressed by the adenoma, which might cause a peripheral regular no-enhancement ring to appear as a result of the interstitial edema and inflammatory exudation [21, 22]. Adenomas of 2 cm or more are accompanied frequently by cystic areas, which reduce the compression force. Therefore, regular high-enhancement rings are observed frequently, which might be caused by the capsule vessels of nodule or blood vessels in compressed adjacent tissue. Nodular goiter nodules with cystic degeneration also display peripheral regular high-enhancement rings. Peripheral irregular high-enhancement and no-enhancement rings tend to be found in nodules between 1 and 2 cm. However, no significant difference in nodules size was found between these two types of irregular rings (*P* > 0.05). The peripheral irregular ring characteristics might be explained by the peritumoral immune response during the invasion process of cancerous cells [23, 24], which might reflect the course of the disease. Peripheral rings on CEUS can provide more useful, additional information, especially for the range of lesions with obscure boundaries on gray-scale US. The visible range of the malignant lesions was often larger on CEUS than on gray-scale US.

This study also had limitations: 95.2% (40/42) of the malignant tumors were papillary thyroid carcinomas in this study, which we believe was just a coincidence. Other malignant pathological types (such as follicular [25] or anaplastic carcinoma) which may have different CEUS enhancement patterns compared with papillary thyroid carcinoma will be enrolled in our further studies.

In conclusion, a peripheral ring on CEUS is an important characteristic for the differential diagnosis of benign and malignant thyroid nodules although this sign did not exist in all the nodules. A high accuracy for nodules with peripheral rings is obtained by CEUS, which could reduce unnecessary biopsies.

## Figures and Tables

**Table 1:** The internal enhancement patterns of benign and malignant thyroid nodules on CEUS.

Internal enhancement patterns	Benign (*n* = 78) (%)	Malignant (*n* = 42) (%)	*χ * ^2^	*P* value
Homogeneous low enhancement	5 (6.4%)	6 (14.3%)	40.61	≤0.001
Heterogeneous low enhancement	1 (1.3%)	11 (26.2%)		
Homogeneous iso-enhancement	23 (29.5%)	6 (14.3%)		
Heterogeneous iso-enhancement	3 (3.8%)	6 (14.3%)		
Homogeneous high enhancement	9 (11.5%)	5 (11.9%)		
Heterogeneous high enhancement	10 (12.8%)	8 (19%)		
Mainly no enhancement or no enhancement	27 (34.6%)	0		

**Table 2:** Peripheral rings of thyroid nodules on CEUS and the corresponding pathological types.

Pathology	Regular high-enhancement ring *n* (%)	Regular no-enhancement ring *n* (%)	Irregular high-enhancement ring *n* (%)	Irregular no-enhancement ring *n* (%)
*Benign*				
Adenoma	20 (28.6%)	5 (41.7%)	0	0
Nodular goiters	40 (57.1%)	2 (16.7%)	0	0
Hashimoto thyroiditis	8 (11.4%)	2 (16.7%)	0	0
Granulomatous thyroiditis	1 (1.4%)	0	0	0
*Malignant*				
Papillary thyroid carcinoma	0	3 (25%)	13 (100%)	24 (96%)
Medullary carcinomas	1 (1.4%)	0	0	1 (4%)
Total	70	12	13	25

**Table 3:** The conventional US and CEUS findings of thyroid benign and malignant nodules.

	Benign histology	Malignant histology	Total
U(−)C(−)	54	1	55
U(+)C(+)	0	40	40
U(+)C(−)	23	0	23
U(−)C(+)	1	1	2
Total	78	42	120

U = conventional US, C = contrast-enhanced ultrasound. (+) indicates malignant diagnosis and (−) indicates benign diagnosis.

## Data Availability

The data used to support the findings of this study are included within the article.
